# Double Number

**Published:** 1867

**Authors:** 


### CHICAGO MEDICAL JOURNAL.

VOL. XXIV. MAY, 1867. No. 5.

### EDITORIAL.

*Double Number.*—April and May have been merged in one issue in order hereafter to anticipate the month in date of publication. Heretofore the plan has been to publish the JOURNAL at any period during the month whose date it bore. Unfortunately the public have grown into the habit of anticipating the time to come, and look upon anything which bears a present date as already somewhat antiquated. To save the JOURNAL the mortification of even appearing belated, the monthly issue will be published and mailed prior to the expiration of the month preceding its date.

As rapidly as possible various contemplated improvements both in the "getting up" and matter will be introduced, and the editor reiterates his promise to spare no labor or expense in its management. The present number, it will be noticed is materially improved in several respects, being printed upon entirely new type and upon paper imported expressly for its use.

